# Supplementary material for: EvANI benchmarking workflow for evolutionary distance estimation
Source: bioRxiv. 2025 Feb 23:2025.02.23.639716. Preprint. [Version 1] doi: 10.1101/2025.02.23.639716 (PMC11870633; doi:10.1101/2025.02.23.639716)
Supplement: 1 [file NIHPP2025.02.23.639716V1-supplement-1.pdf]

bioRxiv preprint doi: https://doi.org/10.1101/2025.02.23.639716; this version posted February 23, 2025. The copyright holder for this preprint (which was not certified by peer review) is the author/funder, who has granted bioRxiv a license to display the preprint in perpetuity. It is made available under aCC-BY 4.0 International license.

6 Supplementary Figures

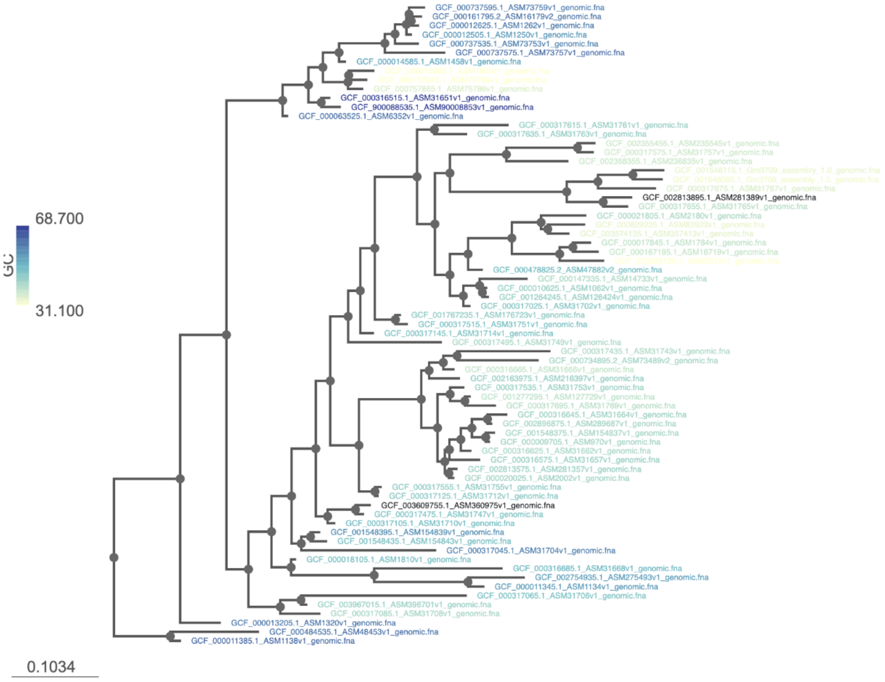

Supplementary Figure 1: The GC content of species in Cyanobacteriota. There is high variation in GC content in Cyanobacteriota, ranging from 31% to 68%.

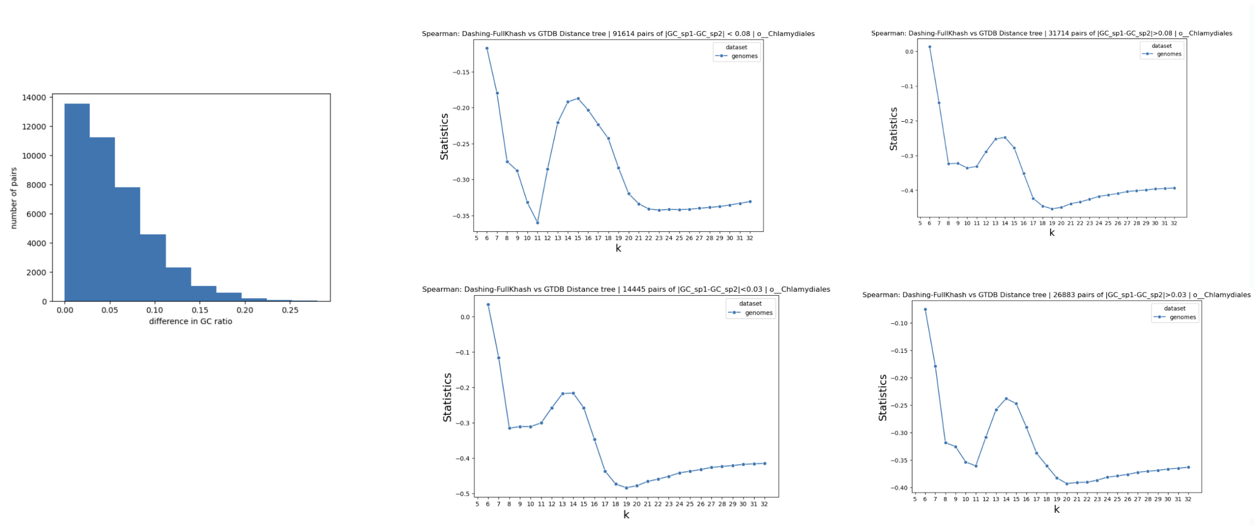

Supplementary Figure 2: (left) The histogram of difference in GC ratio between every species pair. (right) In each subplot, we keep only a portion of pairs. If the GC difference had been the cause (of two minima separate considering all pairs), the sub-figure for pairs with similar GC would not have had two minima. Thus, difference in GC content does not justify the two local minima seen in **Figure 3**.

bioRxiv preprint doi: https://doi.org/10.1101/2025.02.23.639716; this version posted February 23, 2025. The copyright holder for this preprint (which was not certified by peer review) is the author/funder, who has granted bioRxiv a license to display the preprint in perpetuity. It is made available under aCC-BY 4.0 International license.

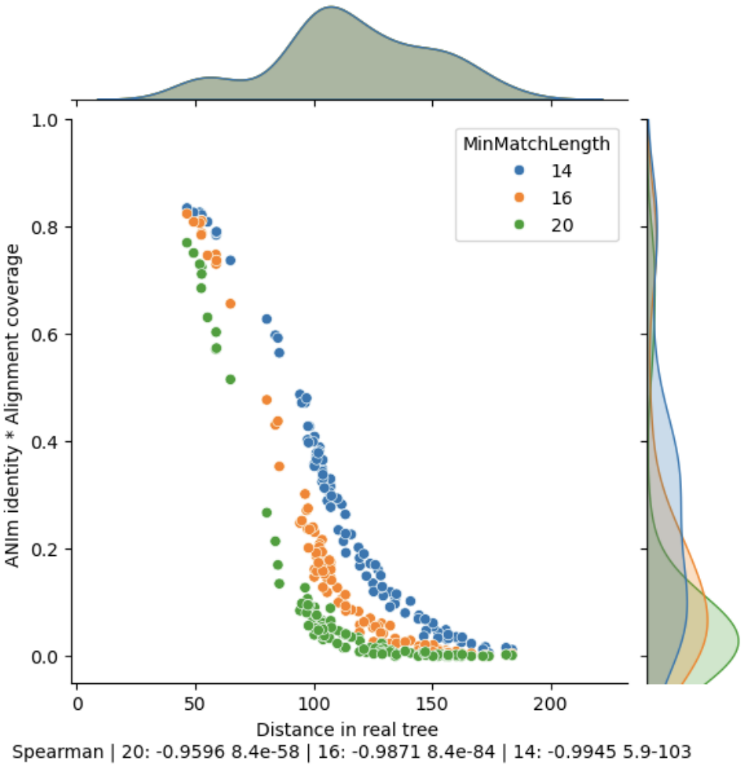

Supplementary Figure 3: Impact of changing the MUMmer parameter minimum match length in distance estimation.

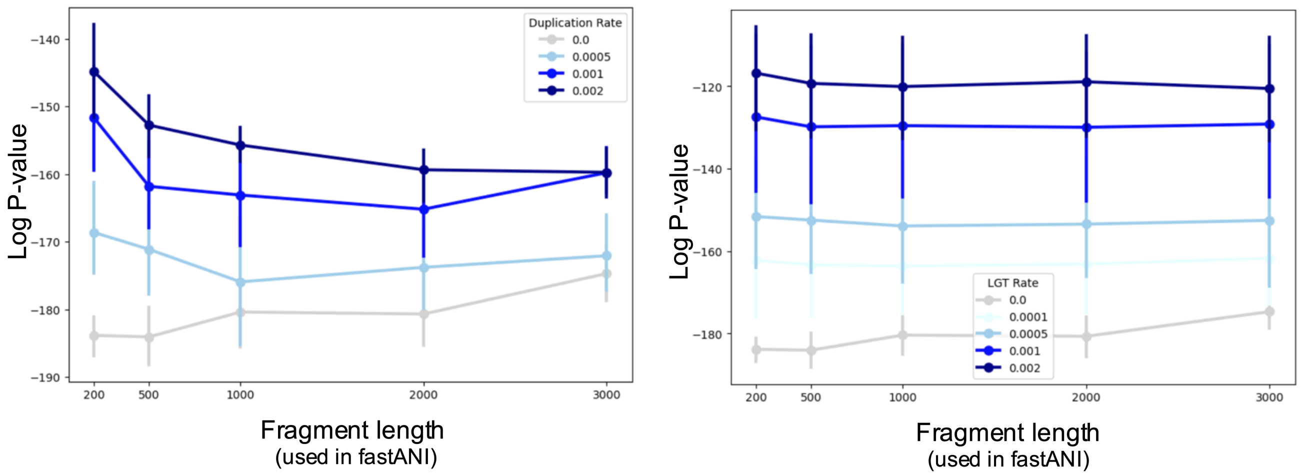

Supplementary Figure 4: Impact of changing fragment length used in FastANI across different simulated data varying duplication and LGT rates.

bioRxiv preprint doi: https://doi.org/10.1101/2025.02.23.639716; this version posted February 23, 2025. The copyright holder for this preprint (which was not certified by peer review) is the author/funder, who has granted bioRxiv a license to display the preprint in perpetuity. It is made available under aCC-BY 4.0 International license.

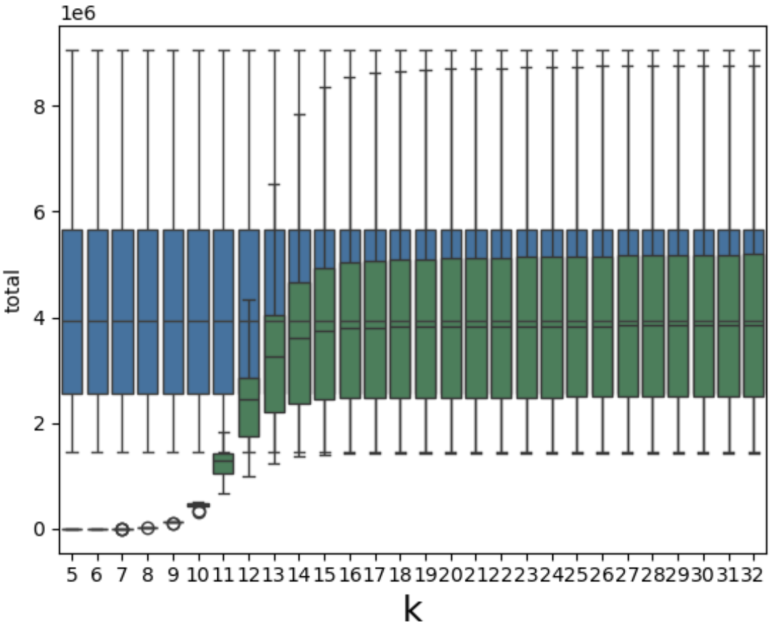

Supplementary Figure 5: (blue) total number of k-mers, which is (almost) equal to the genome size, (green): number of unique k-mers found by the KMC tool. We can see there is a smooth increase in the number of unique k-mers.

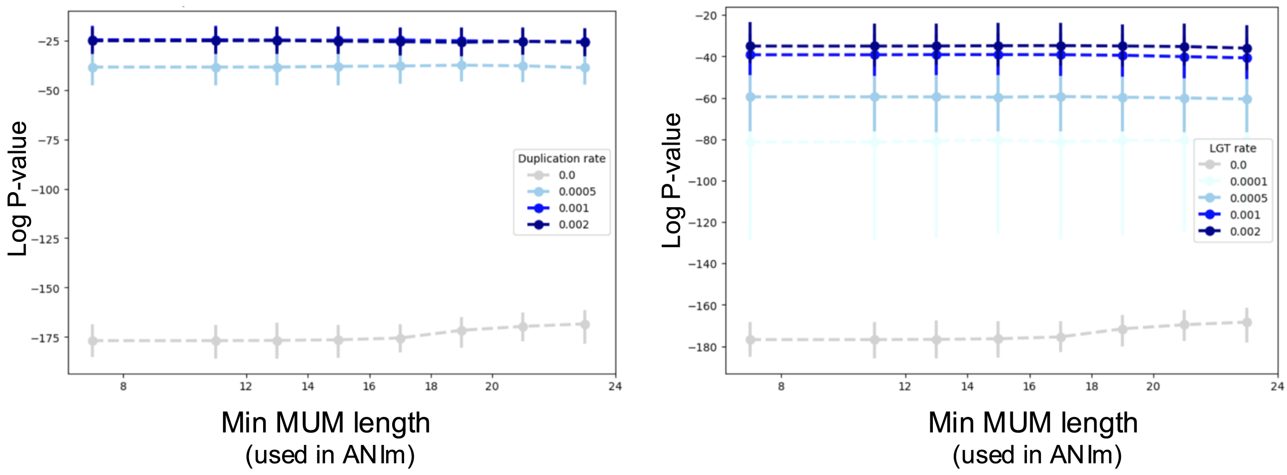

Supplementary Figure 6: The correlation between ANIm∗alignment fraction and tree distance for different minimum MUM length. The alignment fraction here refers to the amount after removing duplicates using `delta-filter -1` to keep only `1-1` alignments. In the last row we reported the alignment fraction before filtering. The simulated results show that the improvement for distant species achieved by weighted ANIm with the alignment fraction does not hold when there is high amount of duplications or LGT.

bioRxiv preprint doi: https://doi.org/10.1101/2025.02.23.639716; this version posted February 23, 2025. The copyright holder for this preprint (which was not certified by peer review) is the author/funder, who has granted bioRxiv a license to display the preprint in perpetuity. It is made available under aCC-BY 4.0 International license.

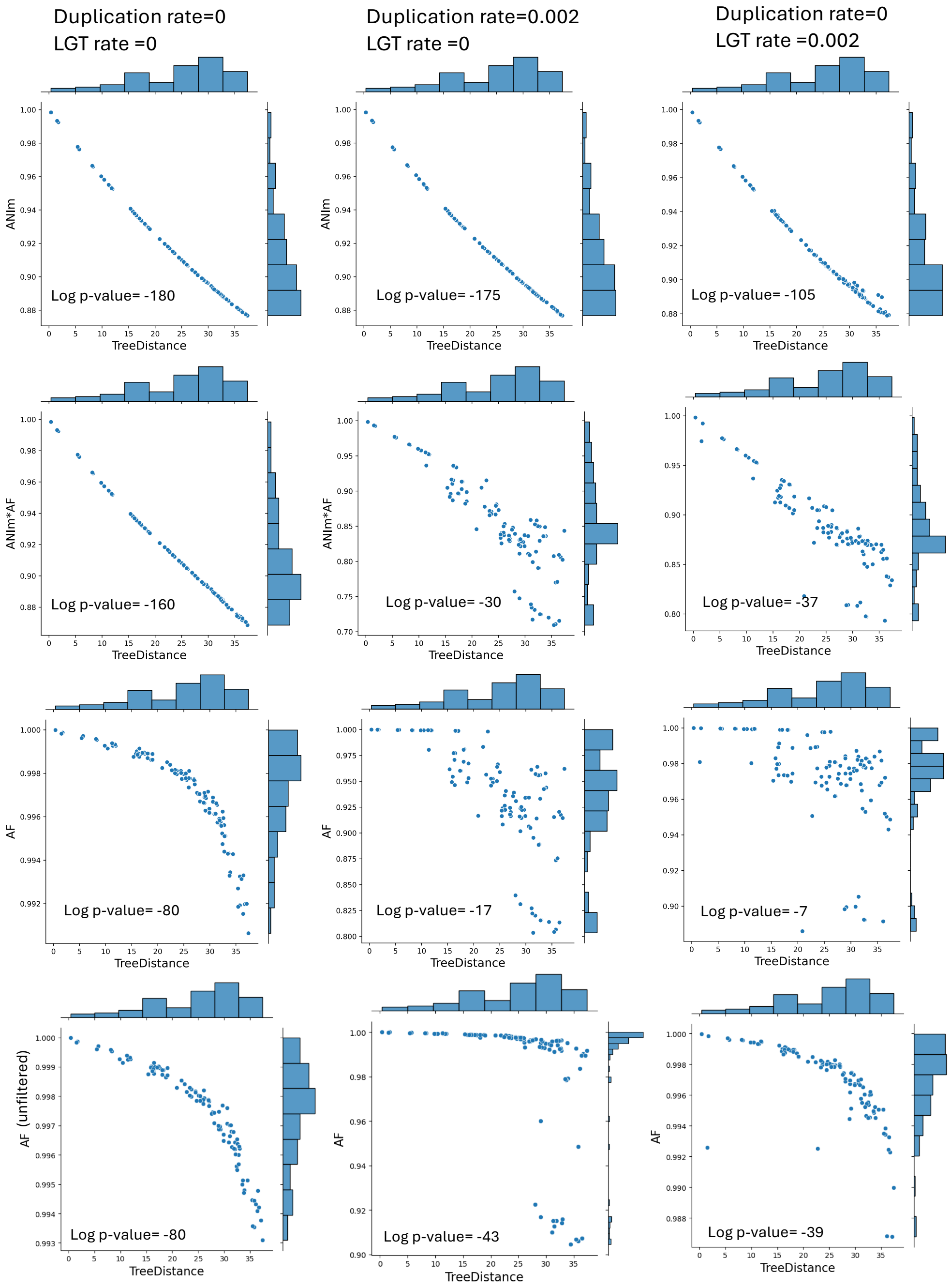

Supplementary Figure 7: The correlation between ANIm∗alignment fraction and tree distance is impacted by the fact that AF is not well correlated with distance when there is higher LGT or duplication. The reported log p-values in the figure are based on the Spearman correlation test. Each point is one of the 105 pairs for 15 simulated genomes. The last two rows correspond to alignment fraction after and before filtering using `delta-filter` to find 1-to-1 alignments. In all figures, NUCmer was executed in MUM mode.

bioRxiv preprint doi: https://doi.org/10.1101/2025.02.23.639716; this version posted February 23, 2025. The copyright holder for this preprint (which was not certified by peer review) is the author/funder, who has granted bioRxiv a license to display the preprint in perpetuity. It is made available under aCC-BY 4.0 International license.

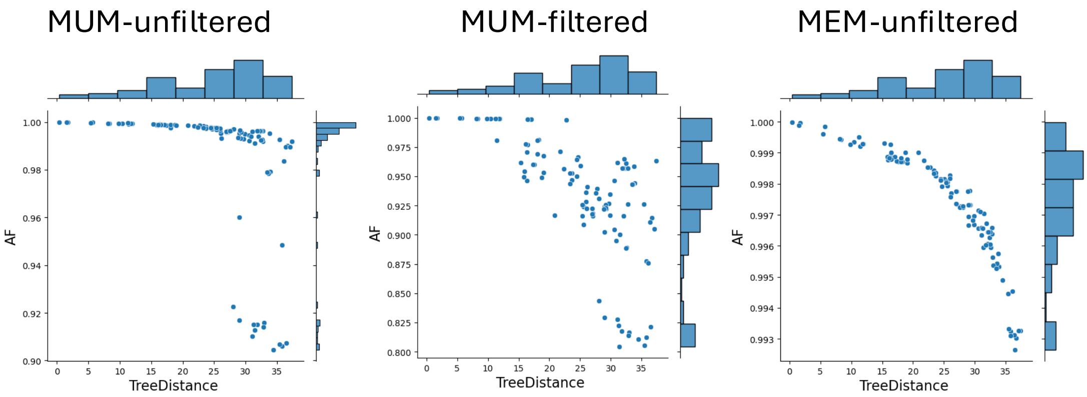

Supplementary Figure 8: The impact of using MEM (maxmatch) instead of MUM with NUCmer in ANIm. Keeping only 1-to-1 alignments (MUM-filtererd) using `delta-filter` also decreases the alignment fraction.

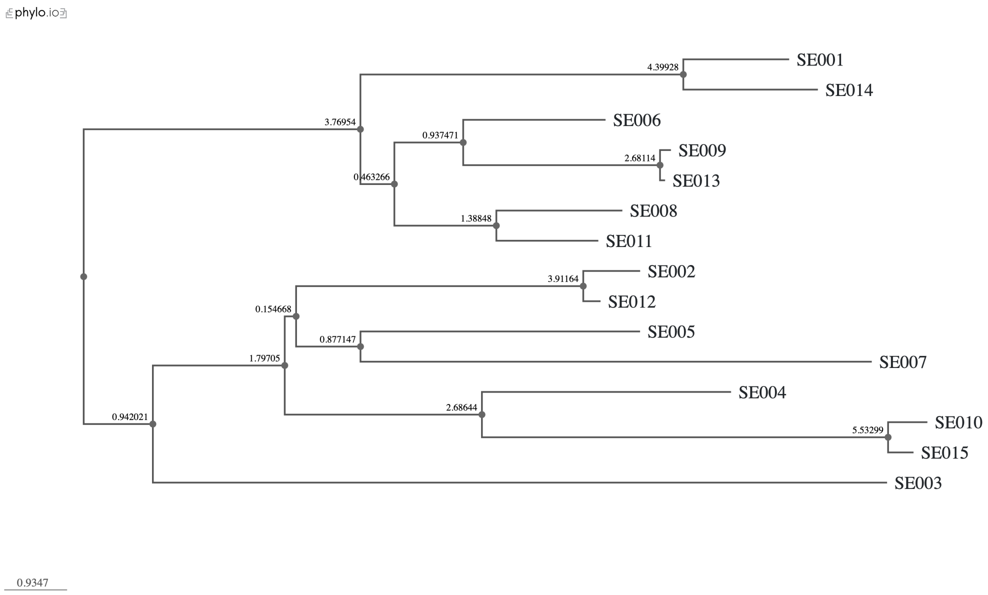

Supplementary Figure 9: An example tree output of the ALF simulator with Mutation Rate of 10. The tree includes 15 species (SE001..SE015).

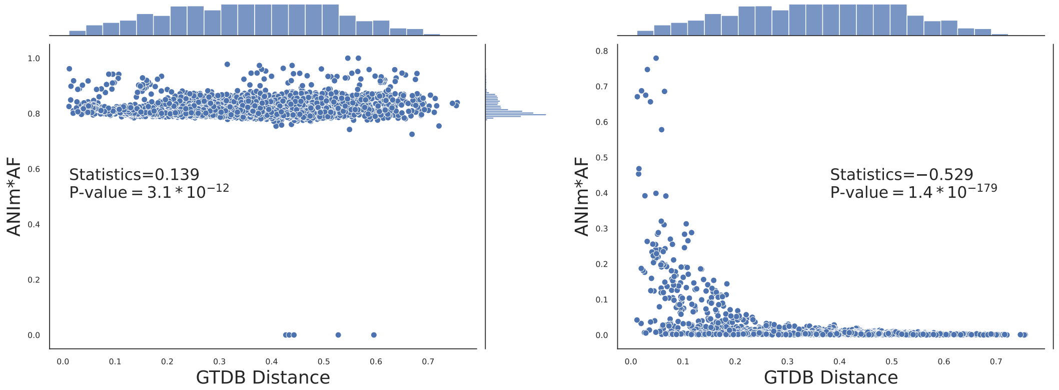

Supplementary Figure 10: Impact of weighting ANIm with alignment fraction (AF) for distance calculation in Cyanobacteraia, similar to Figure 8 but here GTDB tree is used instead of NCBI tree. Here, 71 species are considered and 13 species were discarded due to incompatibility of species names between GTDB and NCBI taxonomy. The same pattern is observed with more pronounced p-values.

bioRxiv preprint doi: https://doi.org/10.1101/2025.02.23.639716; this version posted February 23, 2025. The copyright holder for this preprint (which was not certified by peer review) is the author/funder, who has granted bioRxiv a license to display the preprint in perpetuity. It is made available under aCC-BY 4.0 International license.

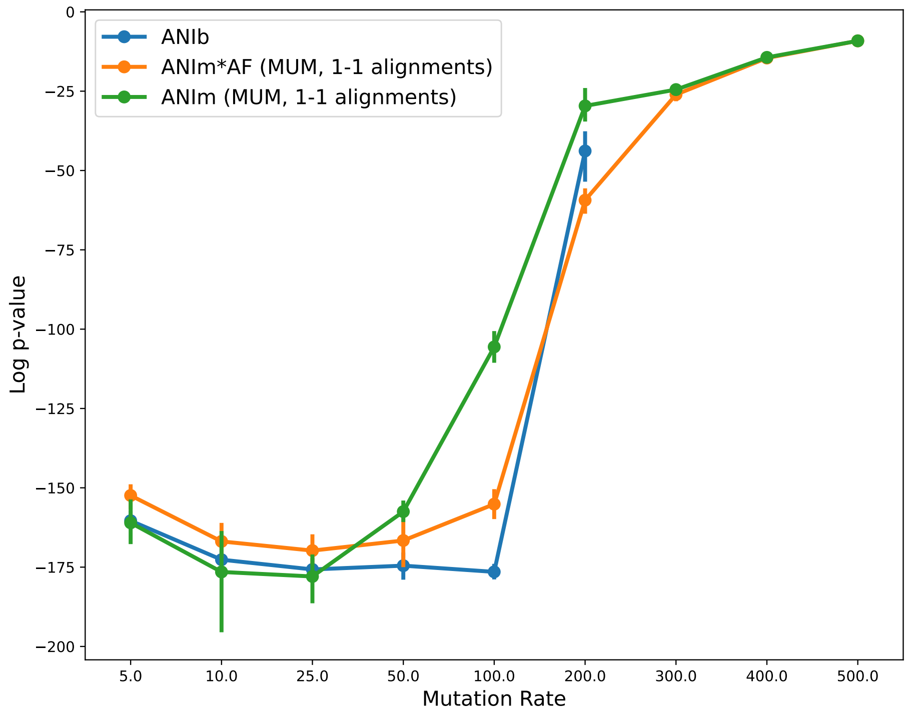

Supplementary Figure 11: Comparing ANIb with ANIm and $ANIm * AF$. Note that when mutation rate is very high (≥ 200), there is not much homology that can be detected by BLAST. BLAST output is empty for these cases when the e-value threshold was e^{-15}.

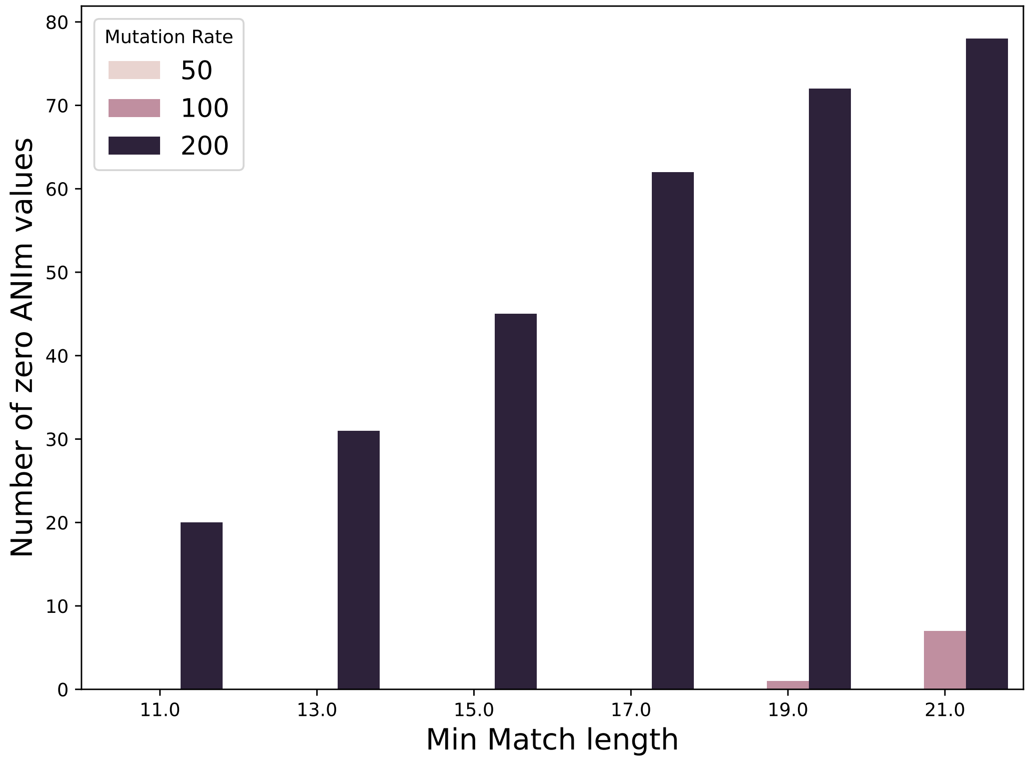

Supplementary Figure 12: Number of zero ANIm values increases by mutation rate (more divergent dataset), affecting the power of ANIm. This could be mitigated by decreasing the minimum match length, ultimately improving rank correlation between ANIm and distance tree (e.g. in **Supplementary Figure 3**).
